# Persistent Changes in Circulating and Intestinal γδ T Cell Subsets, Invariant Natural Killer T Cells and Mucosal-Associated Invariant T Cells in Children and Adults with Coeliac Disease

**DOI:** 10.1371/journal.pone.0076008

**Published:** 2013-10-04

**Authors:** Margaret R. Dunne, Louise Elliott, Seamus Hussey, Nasir Mahmud, Jacinta Kelly, Derek G. Doherty, Conleth F. Feighery

**Affiliations:** 1 Department of Immunology and Institute of Molecular Medicine, Trinity College Dublin, St James’s Hospital, Dublin, Ireland; 2 National Children’s Research Centre, Our Lady’s Children’s Hospital, Crumlin, Dublin, Ireland; 3 National Centre for Paediatric Gastroenterology, Hepatology and Nutrition, Our Lady’s Children’s Hospital, Crumlin, Dublin, Ireland; Karolinska Institutet, Sweden

## Abstract

Coeliac disease is a chronic small intestinal immune-mediated enteropathy precipitated by exposure to dietary gluten in genetically predisposed individuals. The only current therapy is a lifelong gluten free diet. While much work has focused on the gliadin-specific adaptive immune response in coeliac disease, little is understood about the involvement of the innate immune system. Here we used multi-colour flow cytometry to determine the number and frequency of γδ T cells (Vδ1, Vδ2 and Vδ3 subsets), natural killer cells, CD56^+^ T cells, invariant NKT cells, and mucosal associated invariant T cells, in blood and duodenum from adults and children with coeliac disease and healthy matched controls. All circulating innate lymphocyte populations were significantly decreased in adult, but not paediatric coeliac donors, when compared with healthy controls. Within the normal small intestine, we noted that Vδ3 cells were the most abundant γδ T cell type in the adult epithelium and lamina propria, and in the paediatric lamina propria. In contrast, patients with coeliac disease showed skewing toward a predominant Vδ1 profile, observed for both adult and paediatric coeliac disease cohorts, particularly within the gut epithelium. This was concurrent with decreases in all other gut lymphocyte subsets, suggesting a specific involvement of Vδ1 cells in coeliac disease pathogenesis. Further analysis showed that γδ T cells isolated from the coeliac gut display an activated, effector memory phenotype, and retain the ability to rapidly respond to *in vitro* stimulation. A profound loss of CD56 expression in all lymphocyte populations was noted in the coeliac gut. These findings demonstrate a sustained aberrant innate lymphocyte profile in coeliac disease patients of all ages, persisting even after elimination of gluten from the diet. This may lead to impaired immunity, and could potentially account for the increased incidence of autoimmune co-morbidity.

## Introduction

Innate, or unconventional, lymphocytes such as γδ T cells, CD56^+^ T cells, natural killer (NK) cells, invariant NK T (iNKT) cells and mucosal associated invariant T (MAIT) cells, comprise part of a complex immunosurveillance system, where infected, damaged, or otherwise abnormal cells are rapidly recognised and eliminated. Depending on the context of their activation, innate lymphocytes can also display immunoregulatory properties, e.g. invariant natural killer T (iNKT) cells can produce IFN-γ or IL-4 depending on the nature of antigen encountered and the cytokine environment [1]. The role of innate lymphocytes in the pathogenesis of coeliac disease (CD) remain unknown, but it has been reported that NK cells and iNKT cells are reduced in blood and gut of CD patients, and display a diminished capacity for cytokine production [2]. Mucosal associated invariant T (MAIT) cells are also implicated in mucosal immunity, recognising and responding to a diverse set of bacterial and fungal antigens, including microbial vitamin metabolites [3–5]. The role of MAIT cells in CD has not been previously investigated however. Infiltration of T cells into the small intestinal epithelium is one of the earliest events in CD development [6]. Both αβ and γδ T cells are present in this infiltrate, but while αβ T cell levels return to normal upon exclusion of gluten from the diet, γδ T cells remain elevated [6–8]. The significance of this and the specific role of γδ T cells in the gut remain unknown. There are 3 main γδ T cell subsets in humans - Vδ1, Vδ2 and Vδ3. Within the peripheral blood, the majority of γδ T cells possess an invariant Vγ9Vδ2 T cell receptor, whereas the Vδ1/Jδ1-encoded chain predominates in healthy gut tissue [9]. The Vδ1 subset is reportedly expanded in the intestinal epithelium in CD [10–14] and expresses NKG2A and TGF-β, suggesting an immunoregulatory role [8], but data regarding other γδ subsets in the intestine is lacking, or contradictory [15–17]. Since murine γδ T cell subsets differ distinctly from human, and the majority of work on γδ T cells in humans involves the Vδ2 subset, clarification and distinction of the roles discrete γδ subsets play is important, particularly if these cells are to be successfully exploited for immunotherapy [18,19]. Phenotypic and genetic analyses indicate that different γδ T cell subsets may have different, perhaps even opposing roles [20], and developmental pathways [21]. 

In this study we used multi-parameter flow cytometry to characterise the frequency and phenotype of a number of novel innate lymphocyte populations in the blood and gut of adult and paediatric patients with CD. By comparing profiles of healthy control donors and CD patients, we were able to identify persistent alterations in innate lymphocyte populations, as a first step toward elucidating the potential roles for these cells in CD pathogenesis.

## Materials and Methods

### Ethics statement

This study was performed in adherence with the Declaration of Helsinki Ethical Principles for Medical Research Involving Human Subjects. The protocol was approved by the Recognised Ethics Committee at Our Lady’s Children’s Hospital, Crumlin (reference GEN/252/12) and the St James’s Hospital and Adelaide, Meath and National Children’s Hospitals (SJH/AMNCH, REC ref: 2010/07/12) Research Ethics Committee, for paediatric and adult studies, respectively.

### Study populations

Blood samples were obtained from 76 adult patients attending the coeliac clinic at St James’s Hospital, Dublin (SJH), and 78 control subjects recruited from healthy, age- and sex-matched research staff. Controls reported no family history of CD and tested negative for tissue transglutaminase antibodies (tTG). Within the coeliac donor cohort, 56 individuals were defined as treated coeliac donors (TCD), i.e. adhering to a gluten free diet, with accompanying tTG negative serology for at least one year prior to sample collection. The remaining 20 coeliac patients were classified as untreated coeliac donors (UCD) - a population of tTG positive patients on a gluten-containing diet. Duodenal biopsies were obtained from 8 TCD, 6 UCD and 7 control subjects. The control cohort comprised patients undergoing routine endoscopy at SJH, who reported no family history of CD, showed minimal histological inflammation, and tested tTG negative. Blood samples and duodenal biopsies were also obtained from 22 paediatric UCD and 38 age- and sex-matched control patients undergoing endoscopy at Our Lady’s Children’s Hospital Crumlin (OLCH).

In all cases, CD diagnosis was confirmed according to clinical histological and serological analyses. Patients with a known history or subsequent diagnosis of inflammatory bowel disorders were excluded from recruitment/analysis.

All samples were collected with the informed consent of individuals over 18 years of age, or with assent of those under 18 years with consent from guardians, as appropriate.

### Sample collection

Blood was harvested in EDTA and serum Vacutainer tubes (BD Biosciences).

Duodenal biopsies (3-6 sections) were collected into sterile pots containing calcium- and magnesium-free Hank’s Balanced Salt Solution (HBSS) (Sigma-Aldrich) supplemented with 5% v/v foetal calf serum (FCS) (BioWest). Tissue biopsies were processed within 15 minutes of collection.

### Preparation of intestinal tissue

Small intestinal epithelial and lamina propria cells were released from biopsies as previously described [22]. Briefly, duodenal biopsies were agitated for 1 hour in a shaker at 37°C in HBSS supplemented with 5% v/v FCS, 1 mM ethylenediaminetetraacetic acid (EDTA) (Sigma-Aldrich) and 1 mM dithiothreitol (DTT) (Fisher). The resulting epithelial single-cell suspension was filtered through a 40 µM filter (BD Biosciences), washed in complete RPMI 1640 solution (Gibco, with Glutamax, supplemented with 10% v/v FCS, 100 U/ml penicillin, 100 µg/ml streptomycin, 2.5 µg/ml amphotericin B Fungizone and 0.02 M HEPES). Tissue remaining in the filter was collected into complete cRPMI solution containing 130 U/ml collagenase (Type IV-S, Sigma-Aldrich), and rotated for 3 hours at 37°C. Cells were then filtered, washed and enumerated by ethidium bromide and acridine orange staining. T cells isolated from the intestinal epithelium consistently stained >90% positive for the integrin CD103.

### Antibodies and flow cytometry

Absolute numbers of T cells, B cells and NK cells in unprocessed blood samples were determined by TruCount MultiTEST mAb (BD Biosciences) staining and analysis on a BD FACSCanto flow cytometer. For lymphocyte phenotyping, whole blood was stained directly according to BD FACS Lysing Solution recommendations. Dissociated intestinal cells were resuspended in approximately 50 µl phosphate buffer containing 1% v/v BSA, and 0.02% v/v sodium azide, prior to staining. Fluorochrome-labelled monoclonal antibodies (mAb) used for phenotyping included: CD3-Pacific Blue, γδTCR-PE, Vδ2-PE, CD56-PE/Cy7, Vα7.2-APC, CD161-FITC, CD45RA-FITC, Vα24-Jα18-PE (clone 6B11), CD27-APC (all from BioLegend), Vδ3 (unconjugated antibody, clone 11.5B; Beckman Coulter), Vδ1-FITC (ThermoScientific, clone δTCS1). Vδ3 mAb was conjugated to APC dye using a Lightening-Link conjugation kit (Innova Biosciences, UK). Cells were stained, fixed in 1% paraformaldehyde (Santa Cruz Biotechnology) and analysed on a CyAn flow cytometer (Beckman Coulter). NK cells were defined as CD3^-^/CD56^+^ lymphocytes, CD56^+^ T cells as CD3^+^/CD56^+^, MAIT cells as CD3^+^/CD161^+^/Vα7.2^+^, and iNKT cells as CD3^+^/6B11^+^. 

### Intracellular cytokine analysis

Small intestinal epithelial cells were isolated, washed and resuspended in cRPMI medium, and immediately plated at a density of 1x10^6^ cells per ml. Cells were stimulated with either medium only or 10 ng/ml phorbol myristate acetate (PMA) and 1 µg/ml ionomycin (both from Sigma-Aldrich), and incubated for 4 hours at 37°C, 5% CO_2_ in the presence of 10 µg/ml Brefeldin A (Sigma-Aldrich). Cells were then harvested, stained for cell surface markers, fixed in 1% paraformaldehyde (Santa Cruz Biotechnology), permeabilised using 0.2% w/v Saponin (Sigma-Aldrich), incubated with the following intracellular cytokine antibodies: IFN-γ-Pacific Blue and IL-10-APC (BioLegend), IL-17A-PerCP/Cy5.5, IL-21-PE, and IL-22-PECy7 (eBioscience). Cells were analysed on a CyAn flow cytometer (Beckman Coulter). 

### Statistical analyses

Prism GraphPad software was used for data analysis, and differences in control and coeliac donor data were evaluated using a Mann Whitney U test. Statistical advice was provided for this study by Centre for Support and Training in Analysis and Research (CSTAR), University College, Dublin.

## Results

### Innate lymphocytes are decreased in circulation of adult, but not paediatric coeliac patients

**Figure 1:**
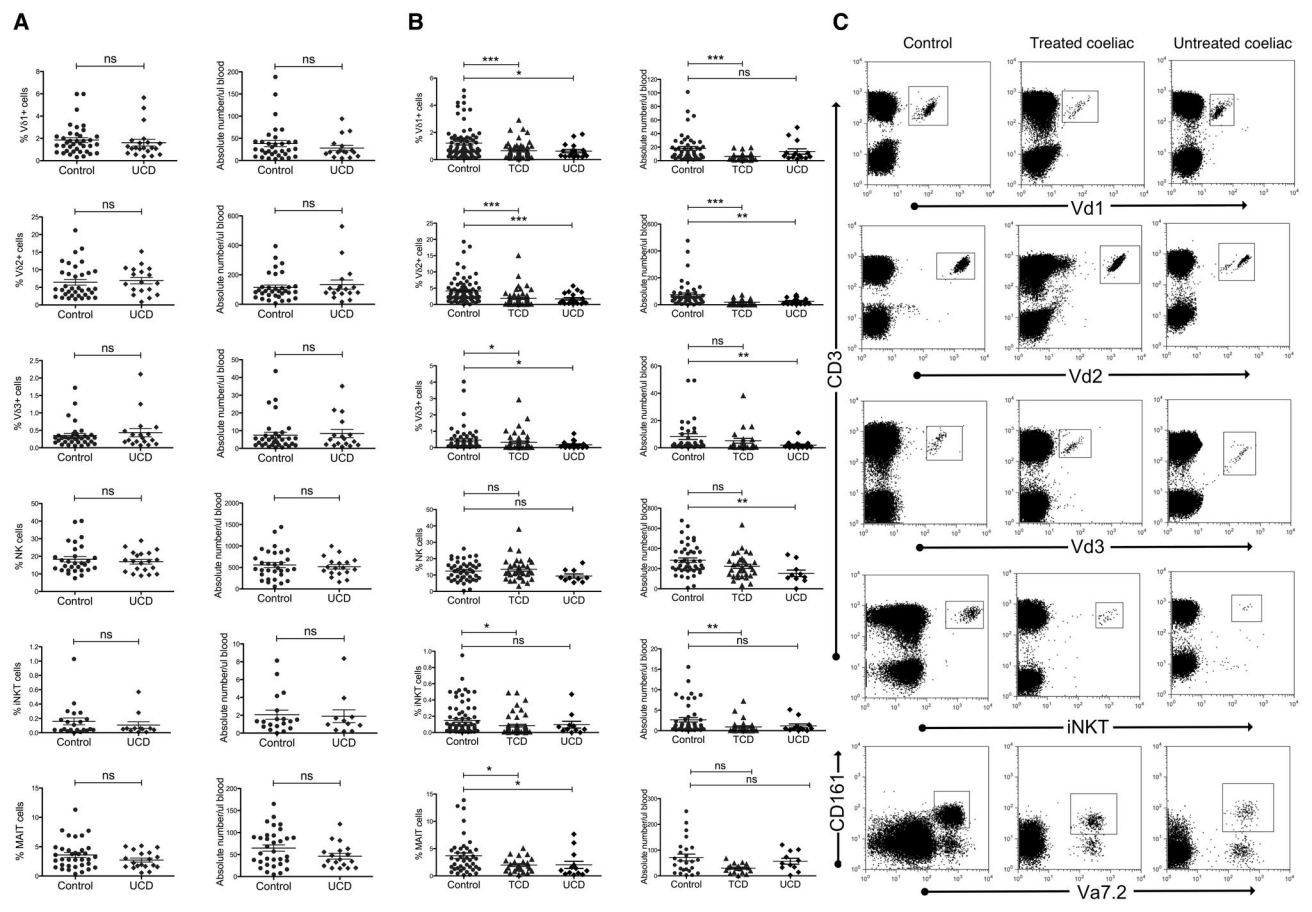
Circulating innate lymphocytes are depleted in adults, but not children, with CD. Scatterplots showing percentages (left column) and absolute numbers (right column) of innate lymphocyte subsets in fresh whole blood, comparing coeliac donors to matched controls for (A) paediatric (n=38 for controls, n=21 for UCD), and (B) adult (n=78 controls, n=56 TCD, n=18 UCD) populations. All subsets are expressed as a percentage of the total T cell population. TCD = treated coeliac donors (on gluten free diet), UCD = untreated coeliac donors (diet contains gluten). Dotplots show flow cytometry data for representative control, treated and untreated coeliac donors (C), where Vα7.2 ^+^ /CD161^+^ populations describe MAIT cells. *P <0.05, **P<0.01, ***P<0.001, error bars show standard error of the mean.

Flow cytometric analysis of whole blood revealed significant decreases in almost all circulating innate lymphocyte subsets for the adult, but not paediatric, CD cohort when compared to normal controls (Figure 1). Adult coeliacs showed marked decreases in all three main γδ T cell subsets (Vδ1, Vδ2 and Vδ3) both in proportion of total T cells and absolute cell number. iNKT and MAIT cells were also reduced in adult CD patient circulation, albeit not as dramatically as γδ T cells. Interestingly, these decreases were observed even when patients were adhering to a GFD, showed negative serology and reported an improvement in symptoms (i.e. treated CD). 

### Innate lymphocyte subset frequencies are perturbed in both adult and paediatric CD patient small intestine

**Figure 2:**
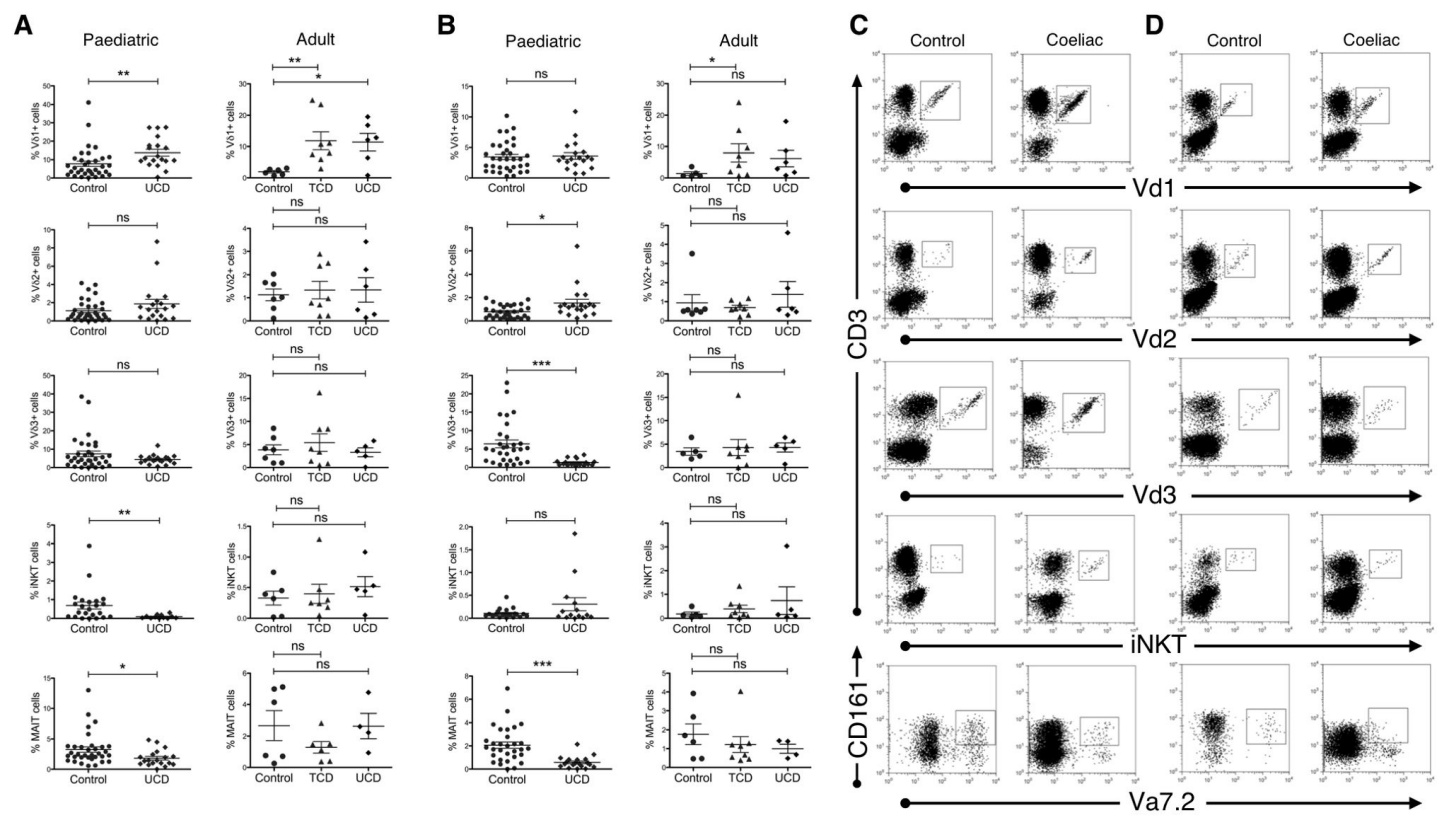
Innate lymphocyte subsets are altered in the intestinal epithelium and lamina propria of both paediatric and adult coeliac donors, compared with controls. Scatterplots showing percentages of small intestinal innate lymphocyte subsets in (A) epithelium, and (B) lamina propria for paediatric (n=34 controls, n=19 UCD) and adult coeliac (n=7 controls, n=8 TCD, n=6 UCD) donors and matched control subjects. All subsets are expressed as a percentage of the total T cell population. TCD = treated coeliac donors (on gluten free diet), UCD = untreated coeliac donors (diet contains gluten). Dotplots show flow cytometry data from gut epithelial cells (C), and lamina propria cells (D) for representative control and coeliac donors, where Vα7.2 ^+^ /CD161^+^ populations describe MAIT cells. *P <0.05, **P<0.01, ***P<0.001, error bars show standard error of the mean.

We noted that Vδ3^+^ cells accounted for the majority of healthy intestinal γδ T cells, in both the epithelium and lamina propria of control donors of all ages, with the exception of the paediatric epithelium, where proportions of Vδ1^+^ and Vδ3^+^ cells were similar (Figure 2). The average Vδ3^+^ cell frequency observed for paediatric control donors was 7.5% in epithelium and 6.4% in lamina propria, compared to Vδ1^+^ cell frequencies of 7.8% and 3.5% respectively. In comparison, in adult control donors, average Vδ3^+^ cell frequencies were 3.9% in epithelium and 3.4% in lamina propria compared to Vδ1^+^ cell averages of 1.9% and 1.4%, respectively. Unlike Vδ1^+^ cells, which were most abundant in the gut epithelium, Vδ3^+^ cells were similarly well represented in both the epithelium and lamina propria. In both adult and paediatric cohorts, we observed that the frequency of Vδ1^+^ cells was elevated in CD gut epithelium, in line with previous literature [10–14]. In contrast to the expansion of Vδ1^+^ cells in CD, Vδ3^+^ cells did not expand, but were instead significantly reduced in the paediatric lamina propria. Intestinal Vδ2^+^ cell frequencies were low in the control donor gut and remained unchanged in CD, except for a moderate increase observed in the paediatric lamina propria. iNKT cells were strikingly reduced in the paediatric CD epithelium when compared to controls, though this trend was not observed in the lamina propria, nor in adult CD patients. MAIT cell frequencies were significantly reduced throughout the paediatric gut, particularly in the lamina propria (a known MAIT cell niche), and a similar trend was noted in adult donors. 

### Small intestinal γδ T cells from paediatric CD patients display a functional effector memory phenotype and are capable of rapid activation upon in vitro stimulation

**Figure 3:**
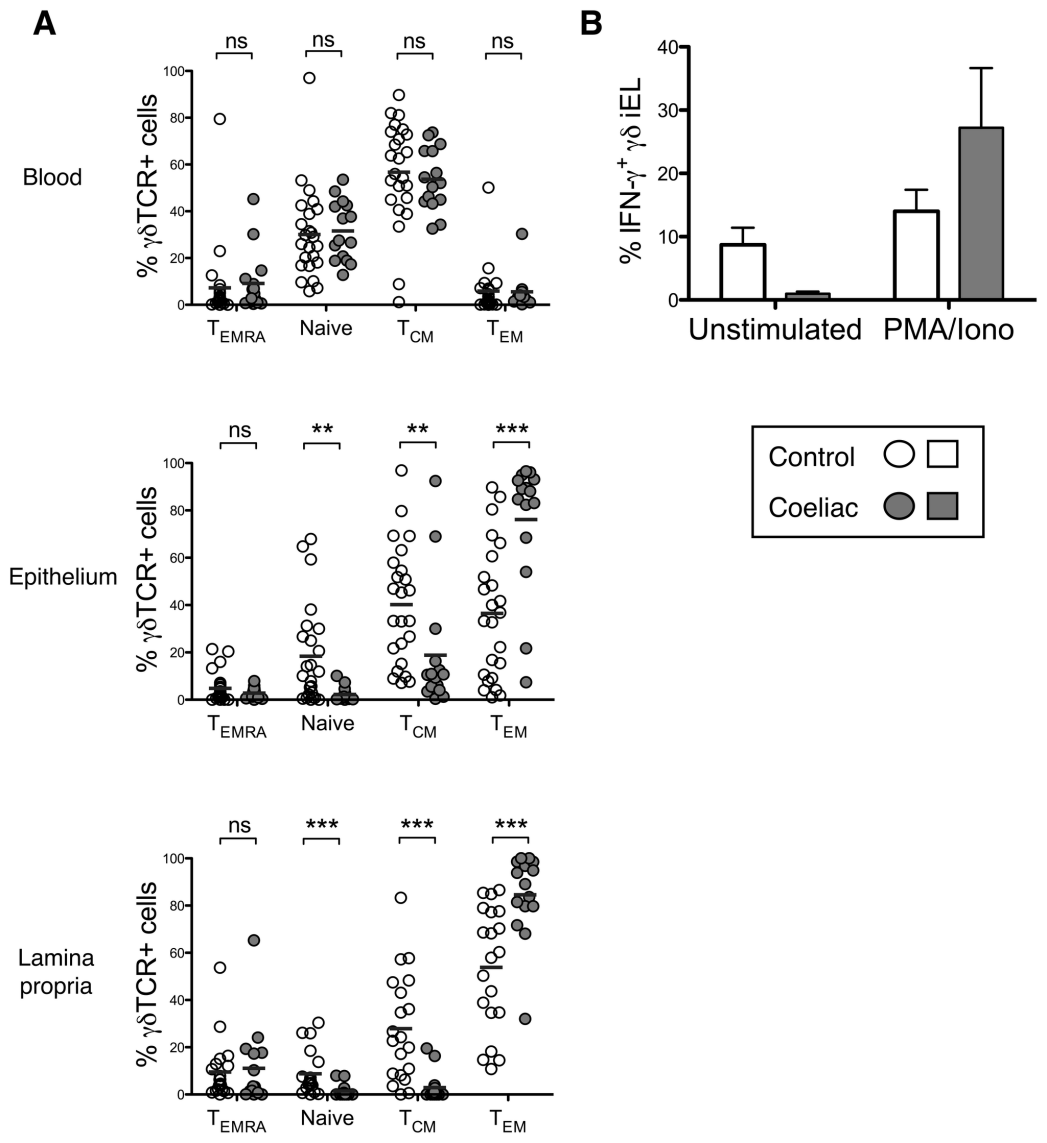
γδ T cells from the coeliac small intestine display a functional “effector memory” phenotype. (A) Differential expression of CD45RA and CD27 by γδ T cells was used to analyse naïve (CD45RA+/CD27+), central memory (TCM; CD45RA-/CD27+), effector memory (TEM; CD45RA-/CD27-) and terminally differentiated (TEMRA; CD45RA+/CD27-) subsets in the blood, small intestinal epithelium and lamina propria from paediatric patients. (B) Expression of IFN-γ by unstimulated and stimulated epithelial γδ T cells, control n=5, coeliac n=4. *P <0.05, **P<0.01, ***P<0.001, error bars show standard error of the mean.

Having observed the abundance of Vδ1^+^ cells in the coeliac gut, we undertook further characterisation of γδ T cells in blood and biopsies taken from paediatric patients (Figure 3). Memory status was evaluated by analysis of CD45RA and CD27 markers expressed by γδTCR^+^ cells in paediatric blood and gut (Figure 3A). Although much variation was seen between individuals, overall frequencies of circulating γδ T cells bearing naïve, central memory (T_CM_), effector memory (T_EM_) and terminally differentiated (T_EMRA_) phenotypes were similar in CD patients and controls. However, in the coeliac gut, strong skewing toward a predominant T_EM_ phenotype (CD45RA^-^/CD27^-^) was observed in the γδ T cell compartment, with a concomitant reduction in the proportion of naïve and T_CM_ cells. This effect was most striking in the lamina propria, where in some donors almost 100% of γδ T cells displayed the T_EM_ phenotype. Similar findings were observed for non-γδ T cells (data not shown). We also analysed the cytokine producing ability of γδTCR^+^ cells from the small intestinal epithelium (γδiEL) stimulated *in vitro* (Figure 3B). Unstimulated γδiEL from CD patients demonstrated a distinct lack of background cytokine production when compared to control γδiEL (as seen for IFN-γ expression, Figure 3B, and also observed for IL-10, IL-17A, IL-21 and IL-22; data not shown). Upon *in vitro* stimulation, γδiEL were capable of rapid and potent upregulation of IFN-γ, at levels similar to those seen for non-γδ T cells (latter data not shown). Taken together, the data provide evidence for the presence of activated and functional γδ T cells in the coeliac gut.

### CD56 downregulation is not restricted to NK cells in the CD gut

**Figure 4:**
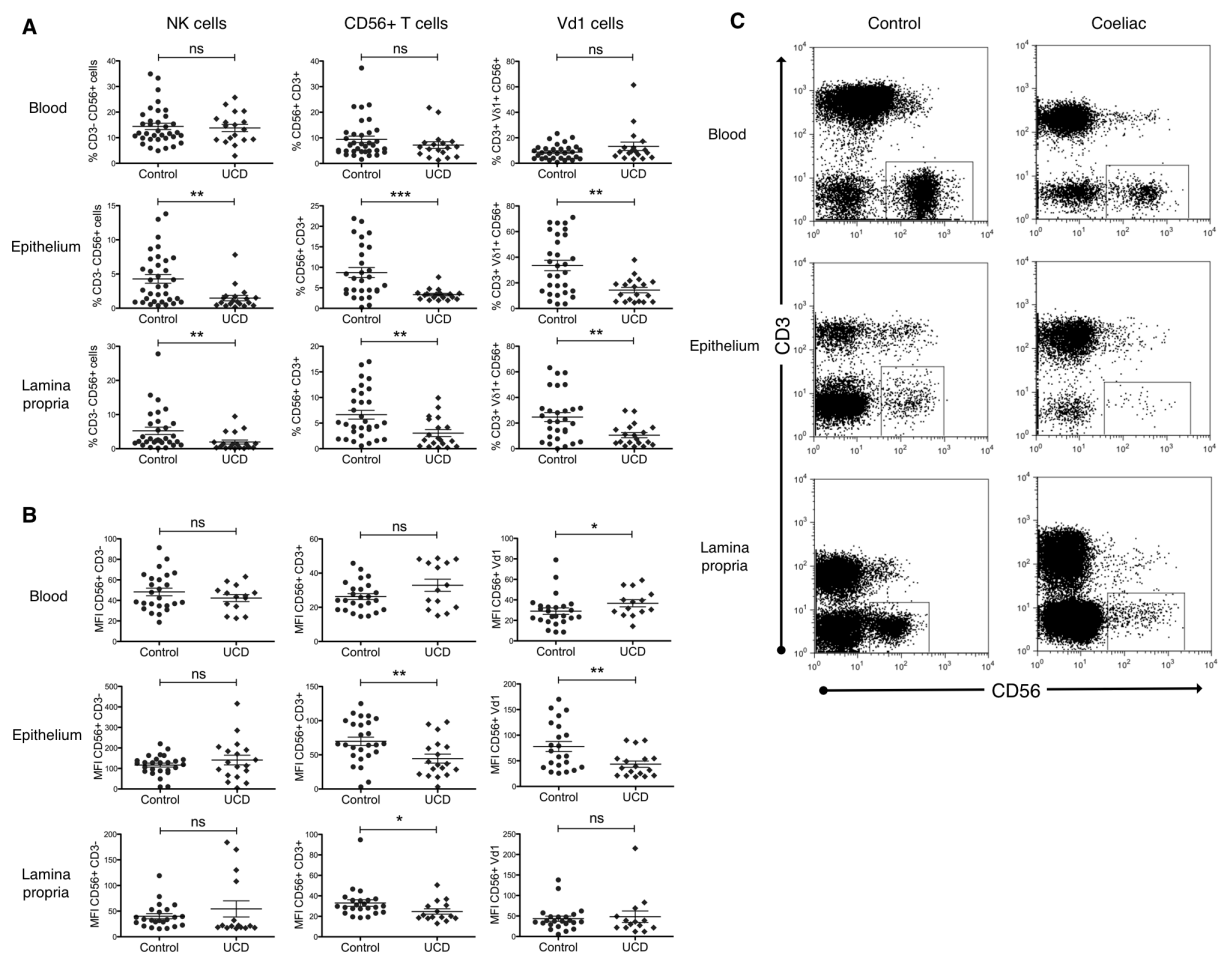
CD56 expression is profoundly downregulated in the coeliac gut, but not blood, both in percentage of cell expression, and expression intensity. CD56 expression was analysed on non-T cells, non-γδ T cells, and Vδ1 T cells in terms of frequency (A) and mean fluorescence intensity (B). Flow cytometry plots are also shown for representative control and coeliac donors (C). For simplicity, data is shown for a paediatric population only, but similar trends were noted in the adult cohort. *P <0.05, **P<0.01, ***P<0.001, error bars show standard error of the mean.

We initially observed that NK cells (defined as CD3^-^/CD56^+^ lymphocytes) were reduced in the small intestinal epithelium and lamina propria of CD patients (Figure 4A, leftmost column, Figure 4C), in agreement with published data [2,23]. However, further analyses revealed that this apparent reduction reflected a more general loss of CD56 expression, by T cells (including Vδ1^+^ cells) as well as non-T cells (Figure 4), and MAIT cells (data not shown). As well as an observed decrease in the percentage CD56^+^ cells (Figure 4A), analysis of mean fluorescence intensity (MFI) further demonstrated an accompanying decrease in CD56 expression intensity, seen for T cell populations but not NK cells, in the coeliac gut (Figure 4B). Similar reductions were noted in adult coeliac patient blood and gut (data not shown), when compared to controls.

## Discussion

In this study we show that the human duodenum hosts several innate lymphocyte populations, including γδ T cell subsets, iNKT cells and MAIT cells, and the relative abundance of these cells is dramatically and persistently altered in CD, for both paediatric and adult cohorts. Using multi-colour flow cytometry, we undertook a simultaneous comparison of innate lymphocyte subsets within the blood, gut epithelium and lamina propria compartments. Our findings provide further detail to support previous studies which showed similar abnormalities in innate lymphocyte populations in CD, specifically, observed decreases in γδ T cells, NK cells and iNKT cells in blood and gut - with the exception of γδ T cells, which are persistently increased in CD small intestinal epithelium [7,8,23,24]. We corroborate the observation that γδ T cell levels (specifically, Vδ1 cells) remain elevated in the coeliac gut even when a patient adopts a GFD and reverts to serological and histological normalcy. Extending these observations, we further show that all major subsets of γδ T cells (Vδ1, Vδ2 and Vδ3), and the newly described MAIT cell subset, are also significantly affected in CD.

We observed a reduction in MAIT cells in both adult and paediatric blood and gut, similar to observations made in the context of HIV [25,26]. In HIV, increased permeability of the gut and associated microbial translocation are thought to drive MAIT cell influx and activation. It is hypothesised that the level of microbial exposure encountered in the HIV-infected gut induces cell death in MAIT cells however, which would account for the observed reduction in MAIT cells in both blood and gut, compared to uninfected controls. A similar scenario likely occurs in the coeliac gut, where gut barrier function is compromised and bacterial overgrowth is common. We also noted a decrease in iNKT cells in coeliac disease. In the healthy gut of children and adults, iNKT cells accounted for <1% of T cells, similar to the frequency noted in blood. It should be noted that in this study iNKT cells were defined solely by phenotypic markers (CD3^+^ Vα24/Jα18^+^). For a definitive iNKT cell characterisation however, α-GalCer/CD1d tetramers are the gold standard. However our observations agree with those of Grose and colleagues, who found that Va24^+^ T cells, Va24 ^+^ /Vb11^+^ T cells and α-GalCer/CD1d tetramer^+^ iNKT cells were numerically deficient and functionally impaired in coeliac disease [2].

It is well documented that Vδ1 cells comprise a major cell population in the normal intestine [14]. However, we report here for the first time that Vδ3 cells also make up a significant proportion of γδ T cells within the small intestine, in healthy donors of all ages. Vδ3 cells accounted for the majority of γδ T cells in healthy adult intestine, in both the epithelium and lamina propria regions, and in the lamina propria of paediatric donors (Vδ1 cells were slightly more abundant in the paediatric epithelium). Thus, while Vδ1 and Vδ3 cells are relatively rare in healthy blood, they represent a major innate lymphocyte population in the healthy human gut. The function and antigen specificity of Vδ3 cells are poorly defined, but our group recently reported that Vδ3 cells respond to glycolipid antigens by killing CD1d^+^ target cells and releasing cytokines that promote T_H_1, T_H_2 and T_H_17 responses [27]. Vδ1 cells have also been shown to recognise sulfatide presented by CD1d tetramers, with some clones demonstrating CD1d recognition in the absence of the sulfatide antigen [28]. Recognition of CD1d by tissue-resident γδ T cell subsets is not surprising given the broad expression of this molecule in the intestine - on dendritic cells, macrophages, B cells and intestinal epithelial cells [29]. Vδ2 cells, on the other hand, are the most abundant type of γδ T cell in blood, but least abundant in the gut and do not appear to respond to CD1d (our unpublished observations). This finding highlights the need for specific identification of γδ T cell subsets in order to more accurately elucidate discrete functions of these different cell types.

Coeliac patients of all ages demonstrated a skewing from a predominant Vδ3 to a predominant Vδ1 phenotype throughout the gut. This was due to an increase in Vδ1 cells and concurrent reduction in Vδ3 cell frequency. It is interesting to note that this increase of Vδ1^+^ T cells in the CD gut, concurrent with a decrease in circulation, directly opposes findings from other inflammatory bowel disorders, where an increase in Vδ1^+^ T cells is noted in blood [30,31], but not within the inflamed gut [10,32]. Our analysis of CD103, a putative gut homing and activation marker [33], revealed similar expression patterns on Vδ1 and non-γδ T cells in blood and gut compartments (data not shown). This similarity held for both coeliac and control donors, except for epithelial Vδ1 cells, which demonstrated elevated CD103 expression in coeliac donors compared to controls. This increase may be explained by TGF-β production by Vδ1 cells [8], which is known to enhance CD103 expression [33]. Further analysis of γδ T cells within the coeliac small intestine revealed a predominant ‘effector memory’ (CD27^-^/CD45RA^-^) phenotype. Although initially this phenotype was used to describe Vδ2 cells in blood [34], the authors’ definition of such cells, ‘abounding at sites of inflammation’ and displaying ‘immediate effector functions’ fit with our findings of an expanded population capable of rapid IFN-γ expression. A corresponding altered phenotype was not observed in blood. We observed a similar effector memory phenotype between γδ and non-γδ T cells (data not shown), in agreement with Kerttula et al., (1995), who also reported an increase in effector memory (defined by CD45RO) Vδ1 cells in coeliac patients, with a similar trend seen for αβ T cells [35]. Despite these observed similarities with inflammatory αβ T cells, evidence points toward an immunoregulatory role for Vδ1 cells [8,36]. We propose a scenario where Vδ1 cells expand in the coeliac gut in response to signals resulting from epithelial cell damage, such as elevated MICA/B expression [37,38]. Activated Vδ1 cells then exert regulatory effects via TGF-β and NKG2A expression, contribute to T cell suppression [8,39,40], anti-tumour immunosurveillance [41], and contribute to gut repair [36,42]. Vδ1 cells lack expression of markers typical of regulatory T cells (our unpublished observations [39,40]), suggesting these cells have a different mode of action to traditional regulatory T cells. Interestingly, Peng et al., (2007) showed that Vδ1 cell suppressive action was abrogated upon TLR-8 ligation. Since TLR-8 is known to recognise viral as well as bacterial components [43], this mechanism could potentially explain the tentative link between rotavirus and CD [44,45].

As well as changes in specific innate subsets, we also noted a profound decline in CD56 expression in the gut of coeliac donors, compared to healthy donors, which extended across all CD56-expressing lymphocytes, including CD3^+^, CD3^-^, all three γδ T cell subsets and MAIT cells (albeit as a preliminary finding for the latter, as only 5 coeliac donors were tested). Loss of CD56 expression was observed both in terms of positive cell frequency and expression intensity. Expression of CD56 by CD3^-^ lymphocytes is most frequently used to define NK cells, whereas expression of CD56 by CD3^+^ lymphocytes identifies a population of T cells capable of potent non-antigen-specific cytotoxicity and cytokine release [46]. Expression of CD56 by CD3^+^ and CD3^-^ lymphocytes has previously been described in the gut and shown to be associated with a cytotoxic, non-proliferative, T_H_1 phenotype [47]. CD56^+^ cells are depleted in inflamed, but not uninvolved, IBD colonic tissue, and in the liver during chronic HCV infection [48]. This decrease has been attributed to concurrent expansion of inflammatory IFN-γ-producing CD4^+^ αβ T cells. A similar scenario could potentially occur in CD, where expansion of gluten-reactive αβ T cells in the small intestine has been demonstrated [49]. The immunological significance of this CD56 downregulation remains to be established, but nevertheless highlights the need for improved phenotypic characterization of NK cells in tissue. Other NK cell markers such as CD16 or natural cytotoxicity receptors (e.g. NKp44 or NKp46) may be useful in this regard [50].

CD is linked to an increased incidence of autoimmune disorders [51,52]. One explanation proposed for this is a dysregulation in communication between innate and adaptive arms of the immune system [53]. In this study we describe significant and persistent changes in innate lymphocyte profiles in CD patients compared to controls. While the function of these cells and their individual contributions to CD pathogenesis remain to be fully elucidated, the diminishment of Vδ2, Vδ3, iNKT, MAIT, NK and other CD56-expressing cells in the coeliac gut is likely to compromise immune defences within the damaged epithelial barrier. Due to persistent elevation within the histologically improved gut after gluten elimination, we propose that Vδ1 cells play roles in regulation and repair within the small intestine, and potentially pose a useful therapeutic target. Our findings further underline the differences in human γδ T cell subsets and highlight the need to discriminate between these subsets in future research. 
